# Methods and Technical Issues for Optimizing the Production of Hydrogels Containing Decellularized Wharton’s Jelly

**DOI:** 10.1021/acsbiomaterials.5c02006

**Published:** 2026-02-03

**Authors:** Anna Chierici, Giovanni D’Atri, Cristina Manferdini, Elisabetta Lambertini, Gina Lisignoli, Roberta Piva, Claudio Nastruzzi, Letizia Penolazzi

**Affiliations:** † Department of Neuroscience and Rehabilitation, 9299 University of Ferrara, I 44121 Ferrara, Italy; ‡ Department of Chemical, Pharmaceutical and Agricultural Sciences, University of Ferrara, I-44121 Ferrara, Italy; § IRCCS Istituto Ortopedico Rizzoli, 18509 Laboratorio di Immunoreumatologia e Rigenerazione Tissutale, 40136 Bologna, Italy; ∥ Department of Chemistry, Materials and Chemical Engineering ″Giulio Natta″, Politecnico di Milano, 20133 Milan, Italy; ⊥ Laboratorio Centralizzato Di Ricerca Preclinica, University of Ferrara, 44121 Ferrara, Italy

**Keywords:** decellularized Wharton’s jelly, extracellular matrix, hydrogels, intervertebral disc, joint degeneration

## Abstract

Bioinspired scaffolds, designed to mimic natural tissue and provide biological cues for tissue regeneration, are becoming increasingly important in the field of tissue engineering. We previously developed hydrogel scaffolds based on alginate and decellularized Wharton’s jelly (DWJ) from an umbilical cord. These scaffolds have proven to be highly effective in promoting the recovery of the lost discogenic phenotype in degenerated intervertebral disc (IVD) cells obtained from patients undergoing discectomy. This prompted us to refine the various steps of the protocol to optimize the development of stable DWJ-based scaffolds with anatomically shaped geometries such as millimeter-scale cylinders (millicylinders) suitable for use in articular cartilage tissue engineering. Particular attention was paid to the handling of the materials used, the reproducibility of data, and the adaptability of the developed system to different experimental needs/conditions, including the transmission of mechanical stimuli and the evaluation of the reactivity of the combined cells. Here, we report the characterization of both the physicochemical properties of the hydrogel produced and its specific biological effects by using IVD cells and macrophages as experimental models. The detailed description of the various steps provides a protocol that aims to facilitate the development of DWJ-based hydrogels that may provide new strategies for addressing joint degeneration.

## Graphical abstract

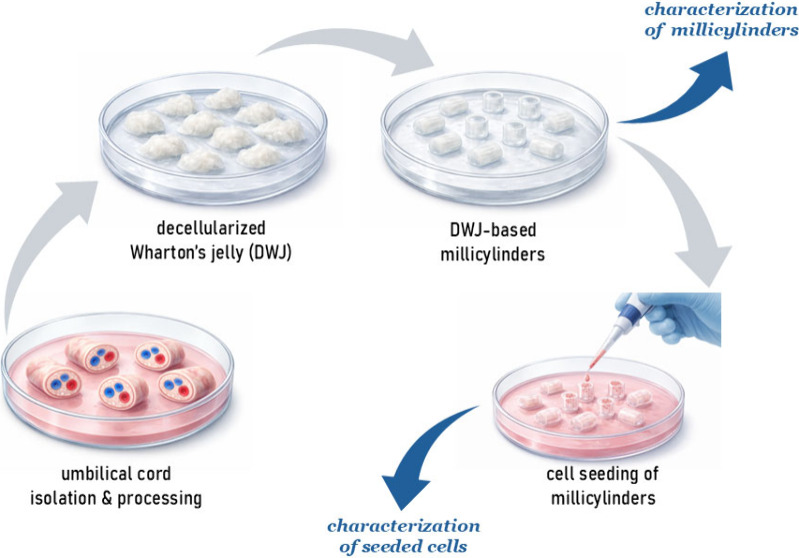

## Introduction

In recent years, there has been growing interest in biological scaffolds, which can be obtained by removing cells from tissues and used for various tissue engineering applications.^1,2^ Through various methods, it is possible to obtain decellularized extracellular matrices (dECM) that can be used as structural and bioactive supports to guide tissue repair/regeneration.^1,2^ Current scientific evidence suggests that dECM is an ideal candidate, when combined with other biomaterials such as alginate, for optimizing the development of multifunctional composite hydrogels.^1–4^ In this context, perinatal tissue-derived dECM has proven particularly promising.^5,6^ Perinatal origin offers in fact a number of practical advantages related to ease of procurement, the abundance of obtainable material, and the absence of ethical concerns. Even more importantly, however, is the biological value of the resulting matrix, particularly rich in structural proteins and growth factors.

We have recently developed millicylindrical scaffolds based on composite hydrogels containing alginate and decellularized Wharton’s jelly (DWJ) for potential use in articular cartilage repair/regeneration.^7,8^ Wharton’s jelly is a gelatinous connective tissue derived from the umbilical cord, rich in collagens, glycosaminoglycans, proteoglycans, growth factors, and signaling molecules that remain unchanged after an adequate decellularization process.^9^ Due to its biological richness and viscoelastic properties, DWJ is an attractive candidate for enhancing the biological functionality of alginate-based constructs. The use of alginate in a composite formulation containing dECM such as DWJ aims to improve its mechanical properties and control its degradation kinetics, without compromising its bioactivity or porosity.^10,11^ Furthermore, the millicylindrical geometry developed for the purpose of producing DWJ-based scaffolds suitable for articular cartilage tissue engineering offers practical advantages in terms of handling, reproducibility, diffusion balance, and adaptability to different experimental and translational contexts.^8^ Interestingly, decellularization itself can improve the biological performance of the Wharton’s jelly by revealing cryptic extracellular matrix ligands and concentrating retained vesicles and growth factors.^5,12,13^ Overall, the composite millicylinders we produce represent a versatile format, designed to be used both as implantable scaffolds at the injured joint site and as standardized 3D in vitro platforms to study specific cellular behaviors under controlled conditions. Interestingly, there is a strong similarity between the WJ and the ECM of the intervertebral disc (IVD), a fibrocartilaginous joint that connects the bodies of two adjacent vertebrae.^14^ This similarity, due to their role as hydrated and viscous connective tissue, has led researchers to consider Wharton’s jelly as a potential biomaterial for regenerative therapies aimed at counteracting also the intervertebral disc degeneration (IDD).^7,8,15,16^ IDD is caused by a combination of genetic and environmental factors that can lead to back pain, a condition that significantly impacts quality of life and represents a significant socioeconomic burden worldwide.^17^ In this context, we recently demonstrated the beneficial effect of DWJ when combined with degenerated IVD cells isolated from biopsies of patients undergoing discectomy.^7,8,18^ In the presence of DWJ, IVD cells recover discogenic characteristics, demonstrating that DWJ provides a bioactive microenvironment rich in structural and trophic cues that support cell function and phenotype stabilization.^7,8,18^ These findings have led us to refine the various steps involved in producing these constructs over time, to optimize their use in subsequent functional tests and potential tissue engineering applications. Indeed, despite their great potential, the use of hydrogel-based biomaterials remains an open area of research. The decellularization process, along with the subsequent processes leading to the fabrication of the millicylinders, including hydrogel preparation, cross-linking, sterilization, and storage conditions, significantly impacts biocompatibility, viscoelastic properties, structural integrity, and the formation of a stable 3D construct. Furthermore, when combining hydrogels and cells, it is essential to develop specific protocols that support cell viability, proliferation, differentiation, and ECM production. At the same time, appropriate analysis protocols need to be developed. The purpose of this technical report was to further explore these critical issues and address the challenges that, through the development of high-performance hydrogel-based constructs, could lead to specific solutions for the repair, replacement, or regeneration of cartilage tissue.^19–22^

## 2 Materials

### 2.1 Scaffold Manufacturing and Characterization

*Reagents*: sodium deoxycholate, DNase-I and NaCl were from Merck KGaA (MA, USA). Alginate (IE-1105/500) was from Inotech Biosystems Int (MD, USA); Gelatin (PS 150 7 30) was from Sanofi (Paris, France); BaCl_2_ was from CARLO ERBA Reagents Srl (Milan, Italy). *Equipment*: plasticware (Petri dishes, tubes) was Falcon-Corning (Corning, NY, USA), Glassware (vials, syringes) was from Biosigma S.p.A. (Cona, Venezia, Italy), Transwells and Durapore Membrane Filters from Merck KGaA (MA, USA), Stainless steel net (1 mm mesh), MicroCL 17 Microcentrifuge from Thermo Fisher Scientific (MA, USA), NF 1200 Multi-Purpose centrifuge from Nuve (Ankara, Turkey), BH-EN Class II Vertical Laminar Flow hood from Faster Srl (Milan, Italy), Lyovapor L-200 Freeze-dryer from BUCHI (Flawil, Swiss), T25 Ultra-Turrax Homogenizer from IKA-Werke GmbH & Co (Staufen, Germany), T.ARE Magnetic Stirrer from VELP Scientifica Srl (Usmate, Italy), STR6 Platform Rocker from Stuart scientific (Redhill, UK), analytical balance from Gibertini Elettronica Srl (Milan, Italy), Dino-Lite Digital Microscope (mod. AM73515MZT) from AnMo Electronics Corporation (Hsinchu, Taiwan), PerkinElmer Spectrum 100 FT-IR (MA, USA), MCR 102e Rheometer from Anton Paar GmbH (Graz, Austria), homemade Polydimethylsiloxane (PDMS) master mold; stainless steel scalpel, kelly clamp, spatula, tweezers and scissors.

### 2.2 Cell Culture

*Reagents*: culture media (DMEM HG, RPMI 1640 and Ham’s F12), fetal calf serum (FCS), l-glutamine, antibiotics (penicillin and streptomycin), and 1× Phosphate-Buffered Saline (PBS) were from Euroclone S.p.A. (Milan, Italy). Type IV collagenase, lipopolysaccharide (LPS, cat.no. L6529), Histopaque-1077 solution and Trypsin-EDTA solution were from Merck KGaA (MA, USA). Human M-CSF (cat.no. 300–25), IFNγ (cat.no. 300–02) were purchased from PeproTech (London, UK). *Equipment*: Forma Series II Water Jacket CO_2_ Incubator Model 3100 and MicroCL 17 Microcentrifuge from Thermo Fisher Scientific (MA, USA), NF 1200 Multi-Purpose centrifuge from Nuve (Ankara, Turkey), BH-EN Class II Vertical Laminar Flow hood from Faster Srl (Milan, Italy), SW-20C shaking water bath from Julabo (Seelbach, Germany), Transwells purchased from Merck KGaA (MA, USA) and Plasticware (culture plates, multiwells, tubes) supplied by Falcon-Corning (Corning, NY, USA), stainless steel tweezers and scissors.

### 2.3 Cell Analysis

*Reagents*: Triton X-100 solution, paraformaldehyde (PFA), LIVE/DEAD Viability/Cytotoxicity Kit for mammalian cells (cat.no. L3224), AlamarBlue Cell Viability Reagent (cat. no. DAL1025) from Invitrogen Thermo Fisher Scientific (MA, USA) and Phalloidin CruzFluor 488 Conjugate (sc-363791) from Santa Cruz Biotechnology (CA, USA), agarose from Aurogene Srl (Roma, Italy), paraffin from Leica Biosystems (Nussloch, Germany), hematoxylin and eosin were from Bio-Optica Milano S.p.A (Milan, Italy), xylene and ethanol from CARLO ERBA Reagents Srl (Milan, Italy), and Aquatex (cat.no 108562) from Merck KGaA (MA, USA). PE conjugated antihuman CD80 Antibody (clone REA661) from Miltenyi Biotec (Germany). Antibody capture beads and 70 μm strainers were from BD Biosciences (NJ, USA). Antihuman CD73 and CD90 were from Abcam (Cambridge, UK); multilinker biotinylated secondary antibody and alkaline phosphatase-conjugated streptavidin and fast red were from Biocare Medical (Walnut CreeK, CA, USA). *Equipment*: Zeiss LSM800 confocal microscope from Carl Zeiss Meditec AG (Oberkochen, Germany), Spark Multimode Microplate Reader from Tecan Group AG (Männedorf, Switzerland), BH-EN Class II Vertical Laminar Flow hood from Faster Srl (Milan, Italy), FACSCanto II Flow Cytometer from BD Biosciences (NJ, USA), Eclipse 90i microscope equipped with a CCD camera (DS-5MC USB2) and Software NIS-Elements (Nikon Instruments Europe BV), paraffin embedder and microtome from Leica Biosystems (Nussloch, Germany), stainless steel molds.

## 3 Methods

### 3.1 WJ Decellularization and Processing

WJ was isolated from human umbilical cords, subjected to a decellularization process, and subsequently freeze-dried to ensure its long-term preservation and stability for use in the fabrication of 3D hydrogel constructs (Figure 1A). Human umbilical cords were collected from cesarean deliveries after receiving informed understanding and written consent from the mothers. The study was conducted with the approval of the Ethics Committee of the University of Ferrara and S. Anna Hospital (protocol no. 061199/AOUFe).

**Figure 1:**
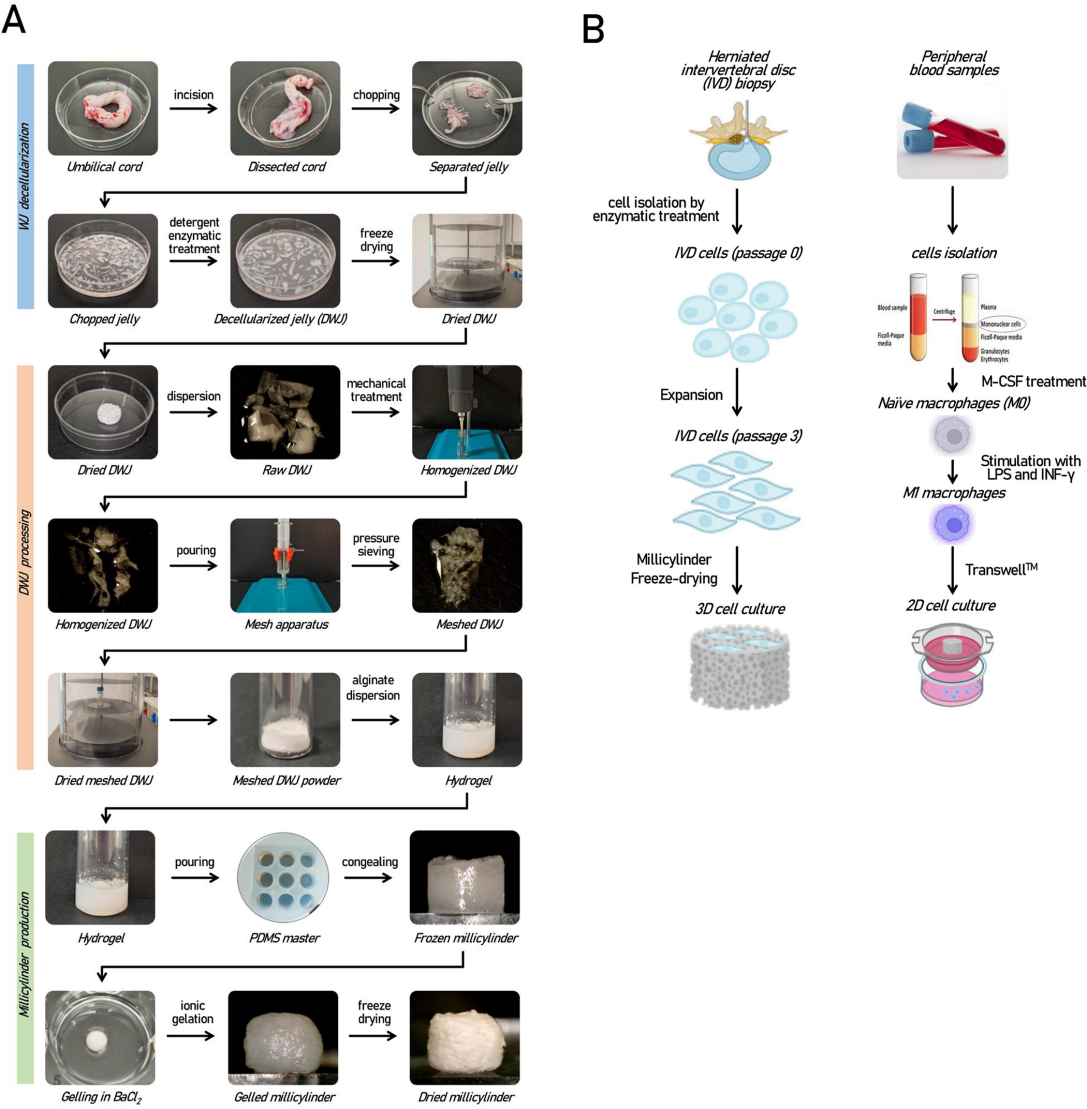
(A) Schematic of the main steps leading from WJ isolation to the fabrication of A075W3 millicylinders composed of alginate and DWJ (see text for details). (B) Experimental cell models: schematic of the main steps for obtaining human IVD cells and macrophages to combine with the millicylinders (see text for details).

#### 3.1.1 Isolation of WJ

Human umbilical cords were processed within 24 h after delivery, rinsed with sterile physiological saline solution (0.9% w/v NaCl), cut into sections (10–12 cm in length) and dissected with a longitudinal cut to remove the blood vessels and to expose the underlying WJ. The soft gel tissue was collected, rinsed in physiological saline solution and carefully minced (approximately 2–4 mm^2^ pieces) (Figure 1A). Ultimately, WJ fragments were stored at 4 °C overnight in physiological saline solution with antibiotics (penicillin and streptomycin).

#### 3.1.2 Decellularization and Freeze-Drying of WJ

WJ was subjected to a detergent-enzymatic treatment (DET) according to a previously validated protocol.^7,18^ This consists of: immersion in sterile deionized water, at 4 °C for 24 h, immersion in a 4% (w/v) sodium deoxycholate (NaDC) solution, at room temperature (RT) for 4 h and, finally, immersion in 2000 kU DNase-I dispersion in 1 M NaCl at RT for 3 h. The decellularized WJ (DWJ) samples thus obtained were then weighed and stored at −20 °C for 24 h, then at −80 °C for 30 min, freeze-dried at 0.6 mbar for 16 h and finally maintained at 4 °C (Figure 1A).

#### 3.1.3 Homogenization of Freeze-Dried DWJ

Freeze-dried DWJ samples were dispersed in 0.1% (w/v) NaDC and mechanically disrupted with a high-speed mechanical homogenizer equipped with a 7.5 mm diameter workhead. The rotor size was chosen to obtain the maximum homogenization capacity on viscous tissues, while the use of a low concentration of NaDC reduces the stickiness of the DWJ and prevents the risk of clogging. The samples were subjected to five consecutive pulses of 5 min each, with increasing speed (from 10,000 rpm to 15,000 rpm) and 30 s pauses between pulses, maintaining them at 4 °C for the entire process (Figure 1A). At the end, the nonhomogenized fragments were discarded.

#### 3.1.4 Production of a DWJ Mesh

The homogenized DWJ samples were pressure sieved with a glass syringe and a sterile stainless steel mesh (1 mm mesh) to remove major fragments and obtain a more homogeneous dispersion, which was collected inside sterile glass vials. To further reduce the stickiness of the DWJ to surfaces and the risk of material loss, each glass tool was rinsed with ethanol and washed in ultrapure water before sterilization. The dispersions were centrifuged repeatedly (at 2000 rpm for 3 min), washed with ultrapure water, then freeze-dried to obtain a homogeneous powder and stored at 4 °C (Figure 1A).

#### 3.1.5 Notes

The critical steps in WJ processing that should not be underestimated are precision dissection to exclude the presence of unwanted blood or epithelial cells; sterile processing; minimizing stickiness to ensure maximum yield, both through the use of low detergent concentrations and appropriate equipment; and optimizing homogenization times to avoid reaching high temperatures that would denature the protein components of the DWJ.

During the processing of WJ, material losses are considerable. From approximately 1 g of fresh WJ, 30 mg of freeze-dried DWJ was obtained. Subsequent homogenization and sieving resulted in an additional loss of approximately 48% ± 8% of the DWJ.

### 3.2 Production and Characterization of Multifunctional Millicylinders

For the purpose described here, DWJ was produced to be used as a component, along with alginate, to create scaffolds with millicylindrical geometry (Figure 1A). As control group, similar millicylinders were produced using commercial animal gelatin instead of DWJ. Gelatin is a natural polymer derived from the partial hydrolysis of insoluble native collagen primarily type I and III which also represent the main collagen components of WJ. Due to its collagen-derived nature, gelatin exhibits excellent biocompatibility, is nontoxic, and rarely triggers significant immune responses or chronic inflammation when implanted.^23^ Furthermore, gelatin is degraded by enzymes present in the body, allowing its progressive replacement with the extracellular matrix secreted by cells. Gelatin, retains the Arg-Gly-Asp (RGD) sequences that promote integrin-mediated cell adhesion, migration, and proliferation. Compared with native collagen, gelatin is cost-effective, easy to handle and it can be processed into various forms, including hydrogels, electrospun fibers, or 3D-printed structures. For these reasons gelatin was chosen as the control material for comparison with DWJ since it is an ideal candidate to mimick the protein components of the ECM (specifically the fibrillar collagen network), allowing for a biologically relevant comparison.

#### 3.2.1 Production Procedures

Sodium alginate or animal gelatin powder were dissolved in pure water, under gentle stirring (200 rpm) for 2 h at 40 °C. Approximately 100 mL of each dispersion was prepared at a concentration of 1.5% (w/v) for sodium alginate and 6% (w/v) for animal gelatin, followed by filtration through membrane filters with decreasing porosity (from 5.00 μm up to 0.22 μm). An additional filtration was performed using a syringe equipped with a 0.22 μm membrane disc filter under a laminar flow hood and the dispersions were stored at 4 °C.

To produce the so-called A075G3 millicylinders, the alginate and gelatin dispersions were preheated to 37 °C and mixed in a 1:1 ratio. The A075W3 millicylinders were instead obtained by diluting the 1.5% (w/v) alginate stock dispersion to 0.75% (w/v) with sterile water and mixing it with the DWJ meshed powder at a concentration of 1 mL per 30 mg (the composition and identification codes are reported in Table 1).

**Table 1:** Identification Codes and Composition of the Produced Millicylinders

identification code	alginate (%, w/v)	gelatin (%, w/v)	DWJ (%, w/v)
A075G3	0.75	3.00	n.p.
A075W3	0.75	n.p.	3.00

100 μL of these formulations were poured into a polydimethylsiloxane (PDMS) master cylinder (5 mm diameter and 1 cm height) and left to gel for 40 min at −80 °C. The solidified millicylinders were gently detached from the silicon master, and ionic gelation of the alginate portion of the polymer mixture was achieved by immersing the samples in a BaCl_2_ solution (1.5% w/v) for 30 min at 4 °C. Finally, the cross-linked millicylinders were rinsed in sterile ultrapure water, transferred to a 96-well plate, freeze-dried, and stored at 4 °C (Figure 1A).

To highlight the importance of the mesh step in producing scaffolds with uniformly distributed DWJ particles, A075W3 millicylinders with homogenized (nonmeshed) DWJ were also prepared by using the same protocol described above.

Fourier transform infrared (FTIR) spectroscopy was performed using a PerkinElmer Spectrum 100 FT-IR spectrometer, equipped with an ATR attachment and Zinc Selenide crystal (ZnSe, code 40326). FTIR analysis was carried out on A075G3 and A075W3 millicylinders to identify chemical compounds and functional groups. Spectra were recorded for alginate, gelatin, DWJ, A075G3 and A075W3 millicylinders in the range 4000–650 cm^–1^.

#### 3.2.2 Water Loss and Water Absorption Capacity, Shrinking and Swelling Ratios

The hydrophilic behavior and dimensional stability of millicylinders (A075G3, *n* = 4; A075W3, *n* = 4) were evaluated by measuring their water loss capacity, water absorption capacity, and shrinking and swelling ratios. To evaluate water loss and shrinking, freshly produced millicylinders were subjected to controlled lyophilization. The weight (*W*_dry_) and length (*L*_dry_) after dehydration were recorded using an analytical balance and image analysis via optical stereomicroscopy, respectively. Water loss (%) and shrinking ratio (%) were calculated as in eqs 1 and 2, respectively:

1 $$\mathrm{water loss}\left(\%\right)=\frac{W_{\mathrm{wet}}-W_{\mathrm{dry}}}{W_{\mathrm{wet}}}\times 100$$

2 $$\mathrm{shrinking ratio}\left(\%\right)=\frac{L_{\mathrm{wet}}-L_{\mathrm{dry}}}{L_{\mathrm{wet}}}\times 100$$

where *W*_wet_ and *L*_wet_ correspond to the weight and length of freshly millicylinders, and *W*_dry_ and *L*_dry_ represent the weight and length after dehydration, respectively. These measurements allowed a quantitative assessment of both water retention and dimensional changes under freeze-drying conditions.

For water absorption and swelling measurements, freeze-dried millicylinders were immersed in water at RT until equilibrium was reached. The hydrated weight (*W*_wet_) and length (*L*_wet_) were then recorded at different time points (5 s, 10 s, 20 s, 30 s, 1 min, 2 min, 5 min, 10 min, 20 min and 30 min). Water absorption (%) and swelling ratio (%) were calculated according to eqs 3 and 4, respectively:

3 $$\mathrm{water absorption}\left(\%\right)=\frac{W_{\mathrm{wet}}-W_{\mathrm{dry}}}{W_{\mathrm{wet}}}\times 100$$

4 $$\mathrm{swelling ratio}\left(\%\right)=\frac{L_{\mathrm{wet}}-L_{\mathrm{dry}}}{L_{\mathrm{wet}}}\times 100$$

The evaluation of these parameters allows us to candidate the produced millicylinders as scaffolds capable of promoting the adhesion, migration, differentiation and proliferation of the cells with which they can be combined, as well as developing a suitable microenvironment for nutrients.

Data are reported as percentage mean ± standard deviation (SD). Unpaired Student’s *t* test was used for direct comparisons; multiple groups were compared by using one-way ANOVA, followed by Tukey’s HSD post hoc test. All statistical analyses were performed using Prism 8 software (GraphPad Software). *p*-values <0.05 were considered significant.

#### 3.2.3 Structural Integrity of the Millicylinder

The structural integrity of millicylinders was evaluated by analyzing the weight of constituents present in the culture medium (released by the millicylinders either in form of solubilized or suspended species) after 7 days into the culture medium. Briefly, freeze-dried millicylinders (A075G3 and A075W3) were accurately weighed (*W*_0_) and individually incubated in 1 mL of medium (DMEM high glucose/F12 + 10% FCS) at 37 °C, to mimic in vitro cell culture conditions. After 7 days, both the supernatant and the millicylinders were collected, freeze-dried and weighed (*W*_7_).

In parallel, the incubation medium was weighed after lyophilization to confirm the mass of solubilized material. A weight percentage 100% was attributed to *W*_0_, and *W*_7_ was expressed as mean weight percentage relative to *W*_0_ (±standard deviation, SD) both for A075G3 (*n* = 3) and A075W3 (*n* = 3). Unpaired Student’s *t* test was used for direct comparisons. All statistical analyses were performed using Prism 8 software (GraphPad Software). *p*-values <0.05 were considered significant.

#### 3.2.4 Rheology

The rheological properties of A075G3 and A075W3 millicylinders were evaluated after 7 days of incubation in 1 mL of standard medium at 37 °C, mimicking in vitro cell culture conditions. Measurements were performed using a rotational rheometer equipped with a plate–plate geometry and a 25 mm diameter plate. The gap between the plates was set to 1.0 mm, and all measurements were performed at 37 °C. An amplitude sweep test was conducted to study the viscoelastic behavior of millicylinders under large deformations and to identify the linear viscoelastic region (LVE). This experiment was conducted at a constant oscillation frequency of 1 Hz, with a shear strain ranging from 0.1 to 1000%, using a logarithmic ramp profile. Data are reported as mean value ± standard deviation (SD) (A075G3, *n* = 5; A075W3, *n* = 3); *p*-values are calculated by Mann–Whitney test and considered significant when <0.05. Statistical analyses were performed using Jamovi Software 2.6.

#### 3.2.5 Notes

Sample preparation with specific component ratios (alginate/gelatin or alginate/DWJ) is a critical step to obtain homogeneous millicylinders and ensure reproducible mechanical and biological properties. Specifically, the cross-linker concentration and exposure time must be properly calibrated to produce constructs with adequate rigidity and structural integrity; furthermore, controlled freeze-drying is necessary to achieve a uniform pore size suitable for subsequent cellular colonization of the scaffold.

Since the well size of the 96-well plate are similar to those of the PDMS mold, freeze-drying the millicylinders within the plate favors the maintenance of a cylindrical geometry.

In rheology, proper loading of the millicylinder onto the Peltier plate is crucial to ensure accurate rheological measurements and reproducible data. The millicylinder volume must exceed this limit to prevent the material from overflowing once the cone is lowered. Inadequate or excessive loading can compromise the uniformity of stress distribution, resulting in measurement artifacts and reduced data reliability.

### 3.3 Cell Culture

Human samples were collected following written informed consent from all participants. All procedures complied with the principles of the Declaration of Helsinki and were approved by the Ethics Committee of the University of Ferrara and S. Anna Hospital (protocol no. 160998 for IVD cells and protocol no. 110952 for monocytes).

#### 3.3.1 Intervertebral Disc (IVD) Cells

Nucleus pulposus (NP) tissue, the soft, gelatinous central portion of the IVD obtained from patients (*n* = 6 mean age 54 years, 3 males, 3 females, Pfirrmann grade 3) undergoing discectomy was dissected, minced in small pieces (2–4 mm^3^), and enzymatically digested with 1 mg/mL type IV collagenase for 5 h at 37 °C (on a shaking plate). Once digestion was complete, the cell suspension was filtered with a 70 μm strainer. The cells were then centrifuged (300 × g, 10 min, RT), resuspended in culture medium (DMEM/F12 containing 10% FCS), and seeded approximately 10000 cells/cm^2^ in polystyrene culture plates. Isolated cells (passage 0) were then expanded to subconfluence, detached by trypsinization, and maintained in culture until passage 3, to obtain IVD cells used for subsequent experiments (Figure 1B).

#### 3.3.2 Macrophages

Peripheral blood (up to 30 mL) was collected from healthy adult volunteers and layered over Histopaque-1077 solution by density gradient centrifugation (400 × g, 30 min, no brake), then washed with 1× PBS and resuspended in RPMI 1640 to obtain PBMCs (peripheral blood mononuclear cells). 3 × 10^6^ PBMCs/cm^2^ were plated in 24-well culture plates and incubated at 37 °C with 5% CO_2_ for 16 h to allow monocyte attachment. Nonadherent cells (lymphocytes) were then removed with 1x PBS by gentle washing. Monocytes were cultured in RPMI 1640 medium supplemented with 10% FCS and 25 ng/mL recombinant human M-CSF to induce differentiation into macrophages (M0), with medium renewed every 2–3 days. After 5 days, the M1 polarization (CTR, control cells) was begun by changing to fresh medium supplemented with 100 ng/mL IFNγ and 100 ng/mL LPS. Subsequently, millicylinders were placed in the upper chamber of 24-well Transwell culture system (0.4 μm pore size) and incubated for 48 h (Figure 1B). After incubation, potential changes in macrophage polarization were assessed by flow cytometry using a specific surface marker (CD80).

#### 3.3.3 Notes

The scarcity of cells in the nucleus pulposus makes it particularly critical to have an adequate number of cells for the planned experiments. Therefore, we suggest using cells from biopsies with a mild degree of degeneration, and expanded in vitro to passage 2 or 3: we have already demonstrated that these cells retain their responsiveness to various biological response modulators.

### 3.4 Characterization of Cells Combined with Millicylinders

#### 3.4.1 Seeding Procedures

A 1% agarose coating in 1× PBS was applied to the bottom of the 96-well culture plate to prevent cell adhesion; feeze-dried millicylinders were then placed on top of the gel coating. Cultured IVD cells (at passage 3) were detached with trypsin and resuspended in filtered medium (with a sterile 0.22 μm membrane filter) at a concentration of 27000 cells/μL. Fifteen μL of the cell suspension (containing approximately 400000 cells) were gently placed on the top surface of the millicylinders and allowed to rehydrate for 2 h at 37 °C. Finally, the samples were added with 100 μL of filtered medium and maintained in standard culture conditions.

#### 3.4.2 Cell Viability and Colonization of Millicylinders

The viability of IVD cells seeded on A075G3 and A075W3 millicylinders and cultured for 7 days, was determined by live/dead fluorescent staining. Briefly, the culture medium was removed, calcein-AM (2 μM) and ethidium iodide (4 μM) staining solution was added directly to the well, and incubated at 37 °C for 30 min. Viable cells showed green fluorescence, while dead cells showed red fluorescence. To determine the ability of millicylinders to support cellular colonization, IVD cells were cultured on millicylinders for up to 7 days, then fixed in a 4% PFA solution, permeabilized with 0.1% Triton X-100 solution and then incubated in the presence of Phalloidin CruzFluor 488 (dilution 1:1000). Fluorescence images were acquired with an inverted microscope (Eclipse 90i, FITC-TRITC filters) equipped with a CCD camera.

#### 3.4.3 Alamar Blue Assay

The proliferative activity of IVD cells seeded and cultured on millicylinders was assessed using the AlamarBlue assay for a 21-day culture period. At predetermined time points (in particular at 1, 4, 7, 10, 14, 17, and 21 days), a ready-to-use resazurin-based solution (diluted 1:20 in DMEM high glucose) was added to the samples. After a 4 h incubation at 37 °C, 200 μL of supernatant was transferred to a 96-well plate and the visible light absorbance was determined by Tecan Spark microplate absorbance reader at 570 nm (resorufin reduced form) and 620 nm (resazurin oxidized form), following the manufacturer’s instructions. Plotted values correspond to the difference in absorbance between the reduced and oxidized forms of AlamarBlue. Data are reported as mean value ± standard deviation. The Shapiro-Wilk test was performed using Jamovi software 2.6 to define normal distribution of the data (A075G3, *n* = 7; A075W3, *n* = 7, for each time point). An Aligned Rank Transform ANOVA (ART-ANOVA) was carried out in R (Google Colab) and p-values were considered significant when <0.05.

#### 3.4.4 Flow Cytometry

Phenotypic analysis of M1 macrophages was performed by flow cytometry. Cells were incubated, for 30 min in the dark, with PE conjugated antihuman CD80. Labeled cells were analyzed using FACS CANTO II compensated using antibody capture beads. Flow cytometry data were analyzed using FlowJo and reported as Mean Fluorescent Intensity (MFI) ± standard deviation (SD). Comparisons were performed by using one-way ANOVA, followed by Tukey’s post hoc test. All statistical analyses were performed using Prism 8 software (GraphPad Software). p-values <0.05 were considered significant.

#### 3.4.5 Histology and Immunohistochemistry

After 7 days of culture, A075G3 and A075W3 millicylinders seeded with IVD cells were fixed in 4% PFA for 40 min and processed for histological and immunohistochemical analyses. Cell-free A075W3 millicylinders, produced using homogenized or meshed DWJ, were fixed in 4% PFA and stained with hematoxylin/eosin to visualize particles distribution.

Histology: Specimens were subjected to graded ethanol dehydration through sequential immersions in 50%, 70%, 80%, 95%, and three changes of 100% ethanol, each for 15 min. Tissue clearing was performed using progressive immersions in ethanol/xylene mixtures (2:1, 1:1, 1:2; 15 min each), followed by three consecutive immersions in 100% xylene for 15 min each. Paraffin embedding was performed by immersing the specimens in graded xylene/paraffin mixtures (2:1, 1:1, 1:2; 30 min each) and then twice in 100% liquid paraffin for 1 h each. Specimens were placed in stainless steel molds, covered with liquid paraffin, and allowed to solidify at 4 °C. Paraffin blocks were sectioned (5 μm thick), deparaffinized, rehydrated, and stained with hematoxylin/eosin for histological evaluation.

Immunohistochemistry: Serial 5 μm sections were incubated for 60 min at RT with mouse antihuman monoclonal antibodies against CD73 and CD90 (both at 5 μg/mL) to evaluate protein expression. After washing, the sections were incubated at RT for 60 min with the primary antibodies, followed by sequential incubation with a biotinylated secondary antibody and alkaline phosphatase-conjugated streptavidin for 20 min each. Colorimetric detection was performed using Fast Red reagent. The sections were counterstained with hematoxylin, mounted with Aquatex aqueous mounting agent and observed under brightfield microscopy. Negative controls and isotype controls were included to confirm staining specificity.

#### 3.4.6 Notes

To prevent cell adhesion to the culture plate rather than to the millicylinders, the wells needed to be coated with an antiadhesive surface treatment. For improved cell penetration into the scaffold structure, freeze-dried millicylinders were rehydrated with a single drop of medium containing the entire cell suspension. The small volume was pipetted slowly onto the upper surface of each scaffold until fully absorbed, followed by a short static period to promote cell infiltration before carefully adding culture medium to avoid cell displacement.

Fluorescent staining provides information exclusively on cells located at the scaffold surface, and the potential interference of the polymers or matrix components with the staining reagents must be carefully evaluated.

Gradual dehydration through increasing ethanol concentrations is essential to avoid tissue shrinkage or distortion, with each immersion performed for the recommended duration to ensure complete water removal while preserving millicylinder morphology. Careful control of the ethanol-to-xylene transition is crucial for efficient tissue clearing; progressively increasing the xylene ratio in ethanol/xylene mixtures allows for a gentle replacement of ethanol with xylene, preventing abrupt changes that could damage the tissue or lead to incomplete clearing. Multiple immersions in 100% xylene ensure complete clearing, which is essential for successful paraffin infiltration, as insufficient clearing may cause embedding artifacts poor-quality sectioning. Temperature and timing during the xylene and paraffin phases must be carefully controlled to maintain the integrity of the hydrogel, as overexposure to these solvents at elevated temperatures can cause the sample to harden or become brittle. During all solvent exchanges, gentle handling is required to avoid damaging the delicate tissue structures of the millicylinder.

## 4 Results and Discussion

### 4.1 DWJ Processing and Millicylinders Production

This report aimed to detail some critical steps for the fabrication of DWJ-based scaffolds, whose pro-discogenic properties we have previously described.^7,8,18^ This was necessary because obtaining further biological evidence required the refinement of additional steps to validate the obtained results and to benefit those approaching similar topics. In addition to the focus on the production of scaffolds composed of a matrix such as DWJ rich in anabolic factors, with a geometry reminiscent of that of IVD (millicylinders), we also aimed to provide useful information to optimize the maintenance of a structure favorable for the transmission of adequate mechanical stimuli to the cells. The importance of adequate mechanical stimuli is well-known for IVD cells, as well as those of all joints, to exert their metabolic and signaling functions.^19,20^

Following the manufacturing steps shown in Figure 1A, two different types of millicylinders were produced: A075G3, composed of 0.75% alginate and 3% gelatin, and A075W3, in which DWJ replaces gelatin at the same concentration.

The scaffolds were produced using a replica molding process with PDMS molds, into which the alginate, gelatin, or DWJ dispersions were poured. After gelation via ionic polymerization, the samples were freeze-dried to obtain porous scaffolds for mechanical testing and ex vivo biological evaluation when combined with cells.

The two types of millicylinders, A075G3 and A075W3, showed a similar macroscopic aspect, as can be seen from the images shown in Figure 2. As is well-known, freeze-drying involves freezing the material followed by sublimation of the solvent under vacuum, producing scaffolds with interconnected porous structures favorable for colonization by cells. This evidence, together with FTIR analysis (Figure 2), confirms that DWJ, from a structural point of view, is an excellent substitute for commercial gelatin.

**Figure 2:**
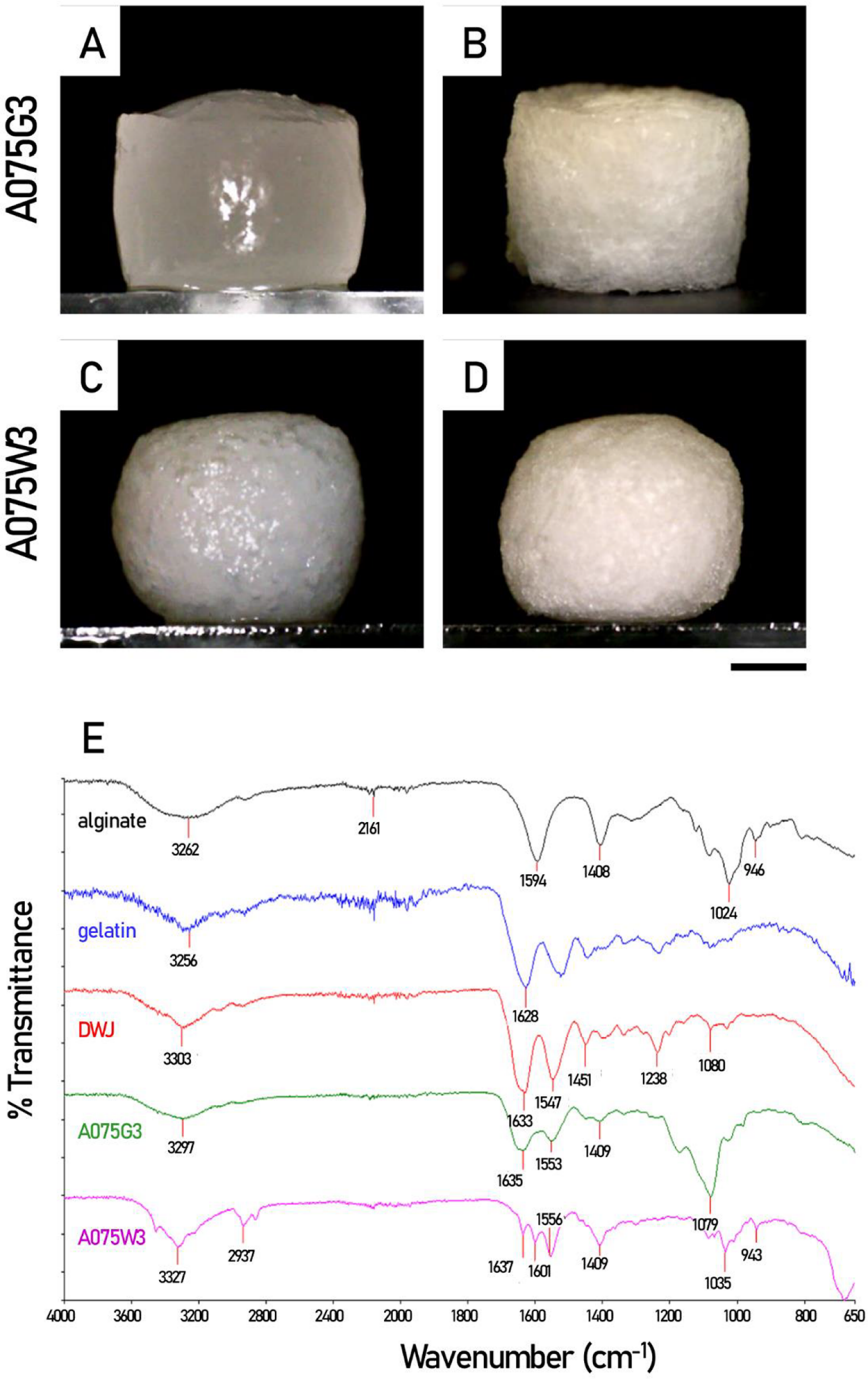
Freshly prepared and ionically cross-linked millicylinders. Representative images of the two types of millicylinders are shown, A075G3 before (A) and after (B) freeze-drying, and A075W3 before (C) and after (D) freeze-drying. (E) FTIR spectra of pure alginate, gelatin powder, and lyophilized DWJ and millicylinders (A075G3, A075W3). The characteristic peaks of the main functional groups are indicated. For the specific composition of the samples see Table 1. Scale bar: 2000 μm.

For the DWJ-based A075W3 millicylinder, the method used for homogenization and filtration through calibrated mesh filters proved effective in obtaining a homogeneous hydrogel structure, as demonstrated by hematoxylin/eosin staining (Figure 3). The development of this phase of the experimental protocol, allowed us to optimize the production of DWJ in a controlled and reproducible manner, to refine the techniques for evaluating the physical properties of the millicylinders and to obtain informative data also from a biological point of view, as reported below.

**Figure 3:**
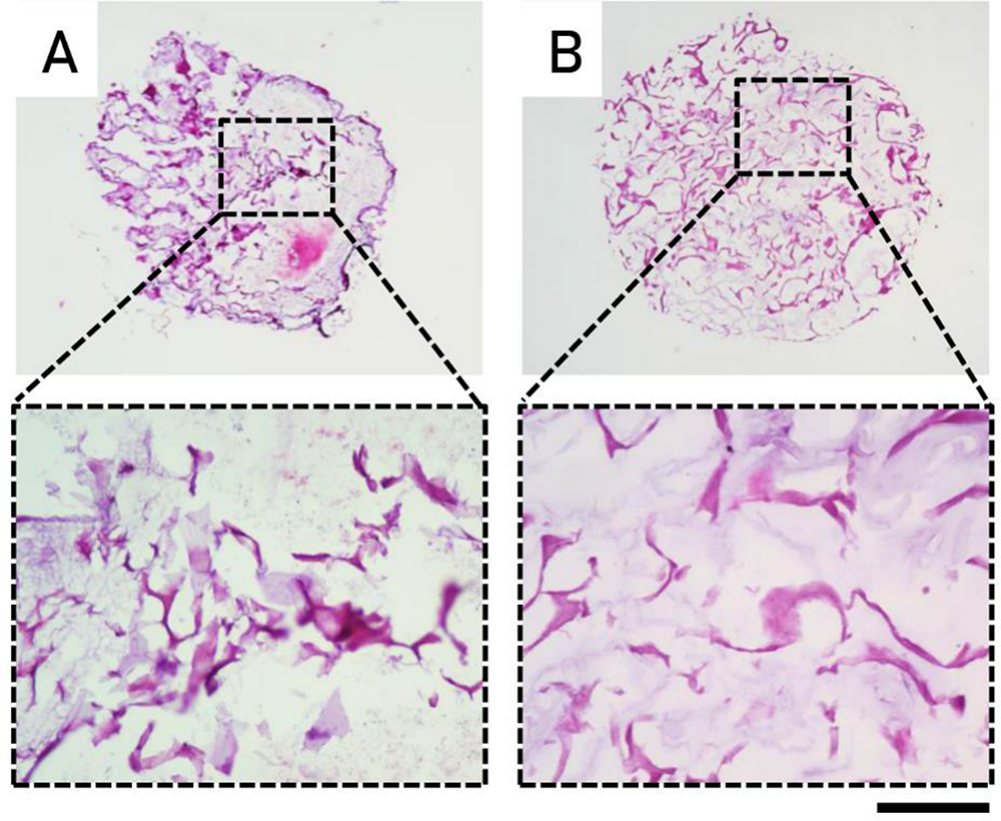
Comparison between A075W3 millicylinders with homogenized DWJ (A) and meshed DWJ (B) in terms of particles size and distribution. Representative hematoxylin/eosin images are shown. Scale bar: 1000 μm (upper images) and 100 μm (lower images).

### 4.2 Water Loss and Water Absorption Capacity, Shrinking and Swelling Ratios

The rehydration and dehydration properties of the millicylinders, including water loss, water absorption capacity, and shrinking/swelling ratios, were systematically evaluated. These parameters are critical for characterizing the scaffold’s hydration dynamics, structural stability, and its ability to sustain cell viability and nutrient diffusion within physiological environments.

To evaluate millicylinders hydrophilic properties, static shrinking/swelling methods are widely employed. In this approach, preweighed, fresh samples undergo controlled freeze-drying until complete lyophilization, and their weight and sizes were recorded (these measurements were expressed as% of water loss and shrinking ratio respectively).

Similarly, to evaluate hydrogel water absorption, preweighed and measured samples were immersed in water for defined periods of time, and their weight and sizes were recorded at regular intervals (these measurements were expressed as water absorption and swelling ratio.

The data collected with these experiments are shown in Figure 4 and can be summarized as follows:

(i) A large fraction of the scaffolds (approximately 90% for A075W3 and 95% for A075G3) consists of water and is removed during freeze-drying (Figure 4A, upper graph);
(ii) A075G3 exhibited approximately a 25% shrinkage in height and 15% in diameter, while A075W3 exhibited a less pronounced shrinkage, approximately 10% and 15% in height and diameter respectively (Figure 4A, lower graph);
(iii) All millicylinders showed rapid hydration kinetics, reaching near-complete water absorption within 30 min for both A075G3 and A075W3, consistent with the high hydrophilicity of the materials used, with comparable water absorption% between the two formulations (Figure 4B, upper graph);
(iv) On the one hand, A075G3 showed a swelling ratio of approximately 5% in height and 8% in diameter after 30 min of rehydration; on the other hand, A075W3 exhibited a minimal swelling ratio: less than 5% after 30 min of rehydration for both height and diameter, resulting in a significantly lower tendency to deform (Figure 4B lower graph; representative images have been reported in Figure 4C).

**Figure 4:**
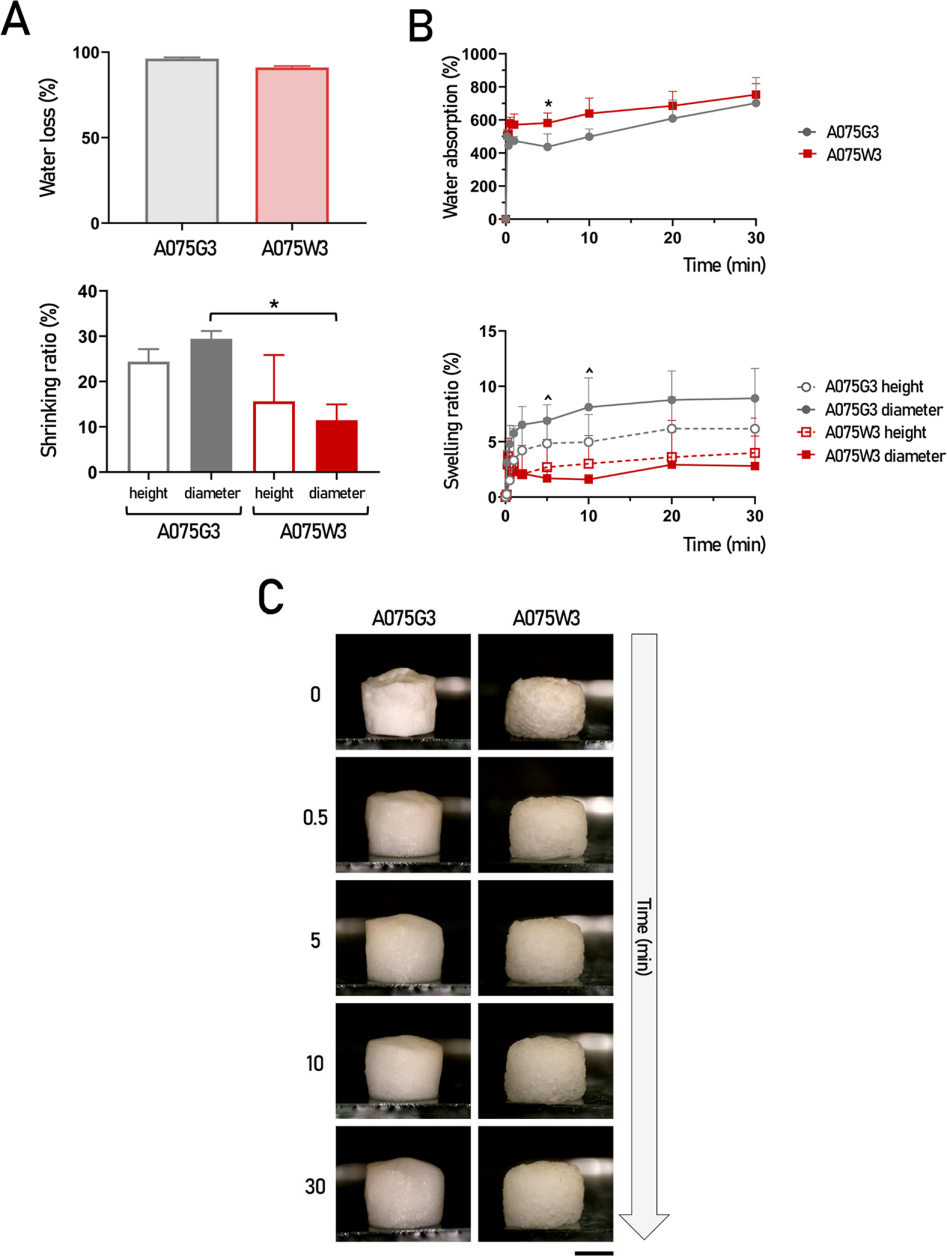
(A) Water loss% and shrinking ratio% (height and diameter) in A075G3 and A075W3 millicylinders subjected to freeze-drying. Values are reported in the graphs as mean percentage ± SD (*n* = 3 for both A075G3 and A075W3). *p < 0.05 (A075G3 diameter vs A075W3 diameter). (B) Water absorption% and swelling ratio% (height and diameter) were evaluated in A075G3 and A075W3 millicylinders subjected to rehydration at different time points, represented by gray circles and red squares, respectively. Values are reported in the graphs as mean percentage ± SD (*n* = 4 for both A075G3 and A075W3). *p < 0.05 (A075G3 vs A075W3); ∧*p* < 0.05 (A075G3 diameter vs A075W3 diameter). (C) Representative images of A075G3 and A075W3 millicylinders during the rehydration process at different time points. Scale bar: 2000 μm.

### 4.3 Structural Integrity of the Millicylinder

Macroscopic and microscopic observation shows that, over time, millicylinders tend to lose their structural integrity by “releasing” constituents, that currently remains to be identified, into the culture medium (either in form of solubilized or suspended species). Notably, the mechanisms causing the structural loss can be multiple and usually characterized by different events, including: (a) biodegradation (or bioresorption), a term that refers to the biological breakdown of the scaffold by local cells and/or enzymes (mainly gelatinases such as MMP-2 and MMP-9); (b) hydrolytic degradation, which describes the chemical breakdown of peptide bonds by water molecules; (c) dissolution (or solubilization), a typical mechanism of low molecular weight hydrophilic molecules present in scaffolds and (d) disintegration/fragmentation, which describes the physical loss of macroscopic integrity where the scaffold breaks into smaller pieces, increasing the surface area/volume ratio and accelerating final resorption.

To determine their structural integrity, freeze-dried millicylinders were systematically weighed before and after 7 days in culture medium. Interestingly, as shown in Figure 5, A075G3 exhibits a significant weight loss of approximately 40%, while A075W3 retains nearly 70% of its original weight (Figure 5). Although this analysis is preliminary and requires further investigation, it suggests that the presence of DWJ in hydrogels can positively influence the maintenance of the structural integrity of scaffolds. This is important, as ideally, implanted scaffolds should lose their structural integrity very slowly, favoring progressive replacement with a newly synthesized matrix. The kinetics of scaffold remodeling also plays an important role in the release of bioactive signals present in the dECM, which in turn can significantly influence the rate of scaffold degradation. It should also be remembered that the structural integrity of the scaffold in vivo depends not only on the composition and structure of the biomaterial itself, but also on the surrounding tissue microenvironment, an aqueous environment in which hydrolysis promoted by enzymatic activity and the mechanical forces to which it is subjected play a key role.

**Figure 5:**
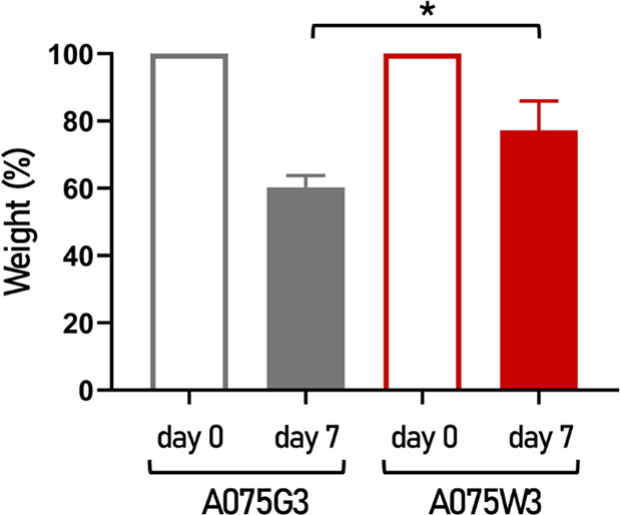
Structural integrity of A075G3 and A075W3 millicylinders. The weight of the freeze-dried samples was recorded before and after immersion in the culture medium for 7 days. The values are reported in the graphs as mean weight percentage ± SD (*n* = 3 for both A075G3 and A075W3). *p < 0.05.

### 4.4 Viscoelastic Properties

The amplitude sweep test was performed to evaluate the viscoelastic behavior of the millicylinders A075G3 and A075W3. The storage modulus (*G*’) as a function of shear strain (%) is reported in Figure 6A. Both formulations showed a plateau region at low shear strain values, indicating the linear viscoelastic region (LVE), followed by a progressive decrease of *G*’ at higher strain values, which is characteristic of the onset of structural breakdown. Notably, sample A075W3 displayed a significantly higher *G*’ compared to A075G3 at 1% of shear strain, as shown in Figure 6B, suggesting that the incorporation of DWJ enhanced the elastic component of the millicylinders and improved its structural integrity under small deformations. This behavior indicates a more stable and interconnected polymeric network, likely due to the presence of ECM-derived proteins that provide additional physical cross-linking and enhance the load-bearing capacity. The higher storage modulus observed for A075W3 supports the hypothesis that DWJ acts as a reinforcing bioactive component within the alginate-based hydrogel, modulating both its mechanical and biological properties.

**Figure 6:**
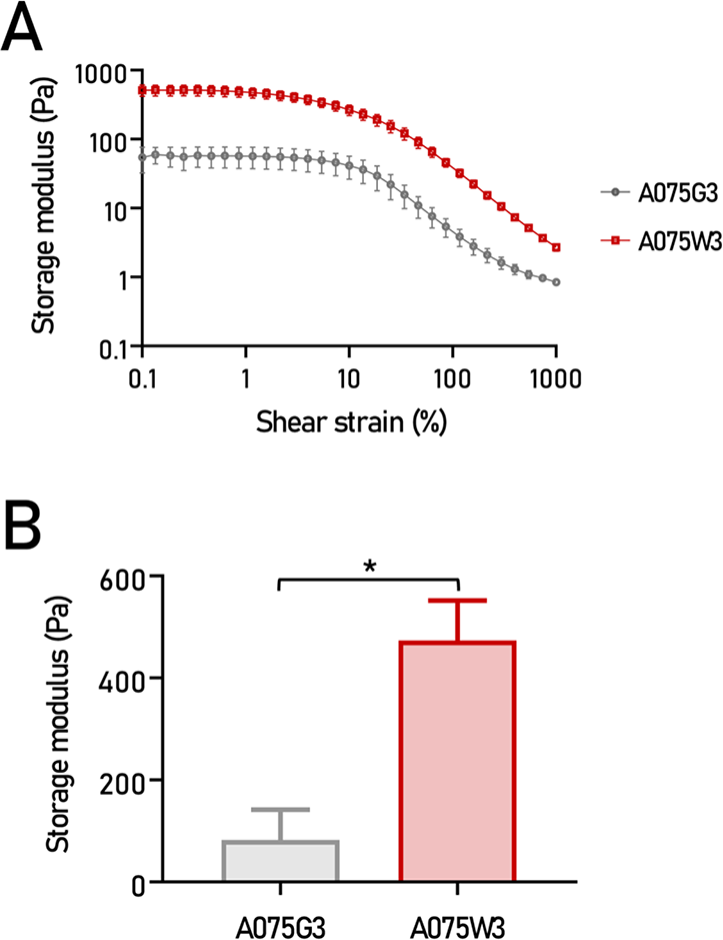
Rheological characterization of A075G3 and A075W3 millicylinders after 7 days of immersion in culture medium. (A) Amplitude sweep, represented with gray circles for A075G3 and red squares for A075W3. (B) Storage modulus (*G*’) at 1% of shear strain for A075G3 (*n* = 5) and A075W3 (*n* = 3). Values are reported in the graphs as mean values ± SD *p < 0.05.

Considering that the elasticity of a cartilage-like tissue such as healthy human NP is approximately 1 kPa, analysis of the viscoelastic properties revealed millicylinder characteristics comparable to those of the target tissue considered here.^24–26^

### 4.5 Evaluation of the Responsiveness of the Cells in Combination with Millicylinders

One of the main challenges in evaluating the biological effects of biomaterials is developing and applying appropriate techniques to monitor cellular responsiveness from different perspectives. This means that each type of scaffold requires significant optimization of the detection systems. In our case, too, we had to overcome critical issues primarily related to the nature of the hydrogel by developing the procedures described in the methodological section. These provided us with interesting data, some of which are described below.

First, without any special precautions, it was possible to apply the live/dead staining with calcein-AM/ethidium iodide, combined with cytoskeletal staining using Phalloidin CruzFluor 488 Conjugate, directly to intact millicylinders. The results indicate that the majority of the IVD cells remain viable after combination with the millicylinders, with negligible uptake of ethidium iodide after 7 days of culture (Figure 7A). As shown in Figure 7B, the cell distribution on the millicylinders surface was homogeneous, suggesting effective colonization of the scaffold matrix by IVD cells. As expected, proliferation assessment performed with AlamarBlue assay, showed that IVD cells receive a low but consistent proliferative stimulus, without significative difference between the two formulations (Figure 8).

**Figure 7:**
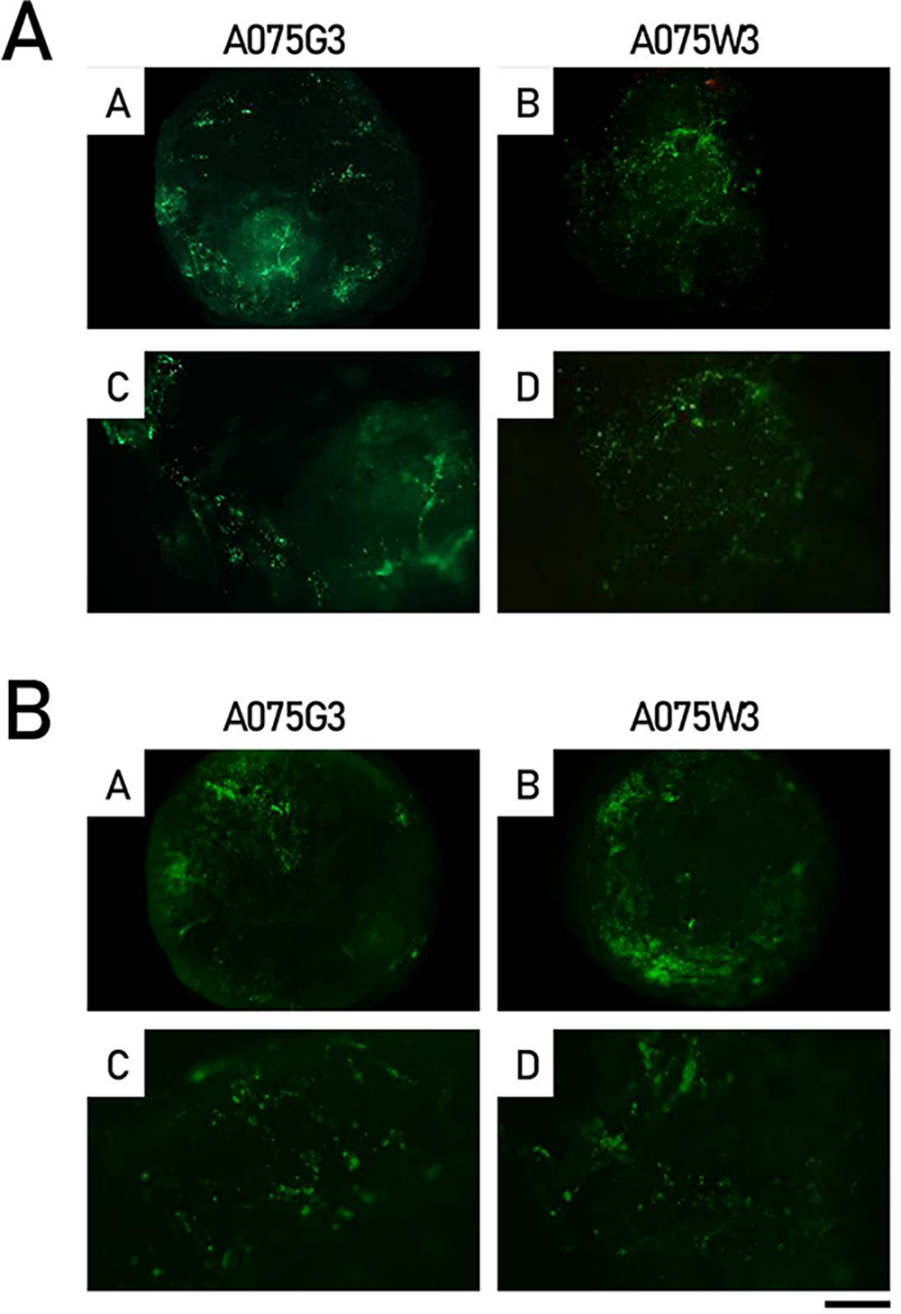
Viability and distribution of IVD cells combined with A075G3 and A075W3 millicylinders for 7 days. (A) Representative images showing Calcein-AM/Ethidium Iodide (merged photomicrographs): viable cells, green fluorescence; dead cells, red fluorescence. (B) Representative images of Phalloidin-stained cells (green fluorescence) distributed in both the central and external zones of the millicylinders. Scale bars correspond to 1000 μm (A, B) and 200 μm (C, D, high magnification images) of each panel.

**Figure 8:**
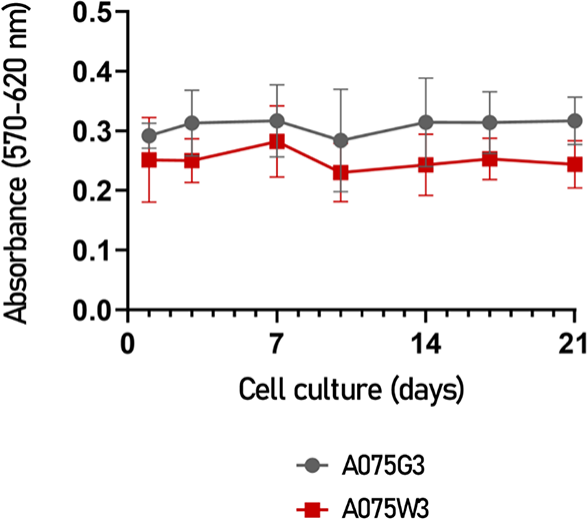
Proliferation of IVD cells combined with A075G3 (gray circles) and A075W3 (red squares) millicylinders for up to 21 days. Cell proliferation was assessed using the AlamarBlue assay. After each time point (1, 4, 7, 10, 14, 17, and 21 days), cells were incubated with resazurin reagent and their absorbance was measured (at 570 nm for the reduced form and at 620 nm for the oxidized form). Values correspond to the difference in absorbance between the reduced and oxidized forms and are expressed as mean ± SD (*n* = 7 for both A075G3 and A075W3).

As described in the Methods section, histological procedures were optimized to treat millicylindrical samples as if they were a kind of tissue, in order to obtain histological sections that could provide reliable data on cellular distribution and specific protein expression.

In particular, hematoxylin/eosin staining of sections obtained from millicylinder A075G3 after 7 days of culture demonstrated that cells preferentially localize at the periphery of a matrix that appears loose and amorphous: therefore, limited cellular infiltration is observed within the millicylinder A075G3. The same analysis performed on millicylinder A075W3 demonstrated that the presence of DWJ leads to a more structured organization of the scaffold where the original fibrillar components of the extracellular matrix from which they originate are visible. Furthermore, the cells colonize the millicylinder A075W3 more uniformly, clustering in a manner that resembles what happens in vivo (see the comparison reported in Supplementary Figure 1). Overall, A075W3 appears to provide a richer biomimetic microenvironment than the A075G3 scaffold, likely offering a more favorable support for host cells (Figure 9). The adequacy of the analysis procedure also allowed us to validate this hypothesis by immunostaining the sections obtained from the millicylinders using antibodies against CD73 and CD90. These are two canonical surface markers of mesenchymal stromal cells, also expressed by disc progenitor cells in IVD tissues.^27^ CD73 is involved in adenosine-mediated signaling and may confer survival and immunomodulatory advantages in the hypoxic and inflammatory conditions typical of degenerated disc tissue. CD90, on the other hand, plays a critical role in interactions with the cellular matrix, in adhesion, and in mechanotransduction signaling. Interestingly, the presence of these two markers was detected particularly in IVD cells combined with A075W3 (Figure 10). This confirmed the potential of the DWJ-containing hydrogel to promote key phenotypic traits of progenitor cells, an important aspect for the development of advanced biomaterials for cartilage and intervertebral disc regeneration.

**Figure 9:**
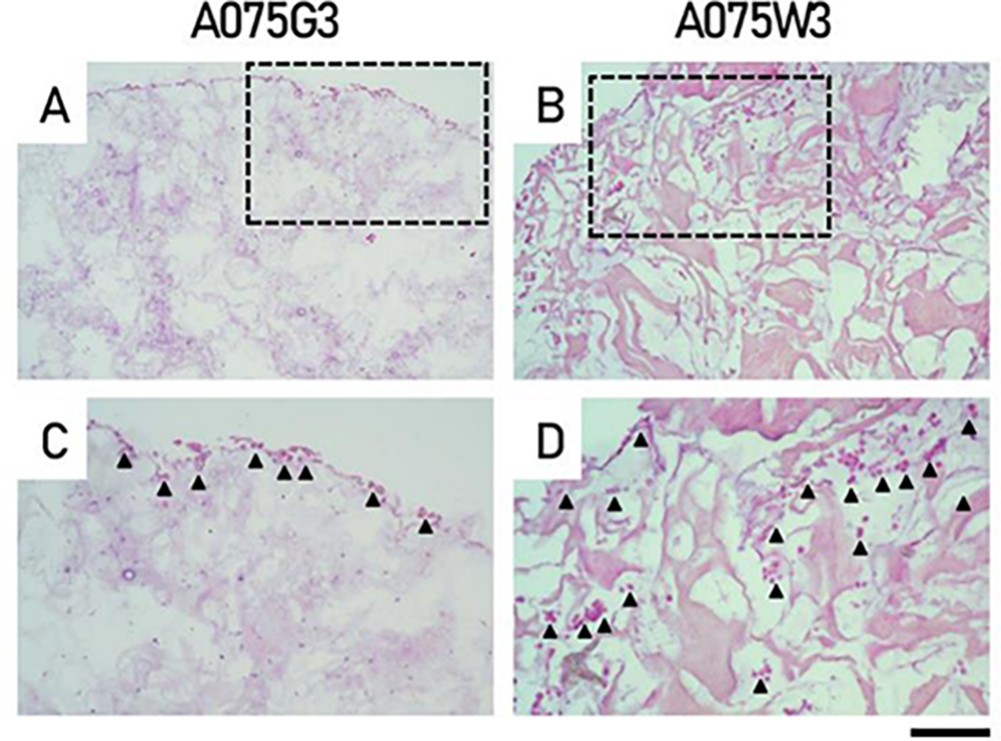
Hematoxylin/eosin staining of IVD cells combined with A075G3 and A075W3 millicylinders for 7 days. Representative images of A075G3 (A, C) and A075W3 (B, D) are reported. Cell distribution is indicated by arrows. Scale bars correspond to 200 μm (A, B) and 100 μm (C, D, high magnification images).

**Figure 10:**
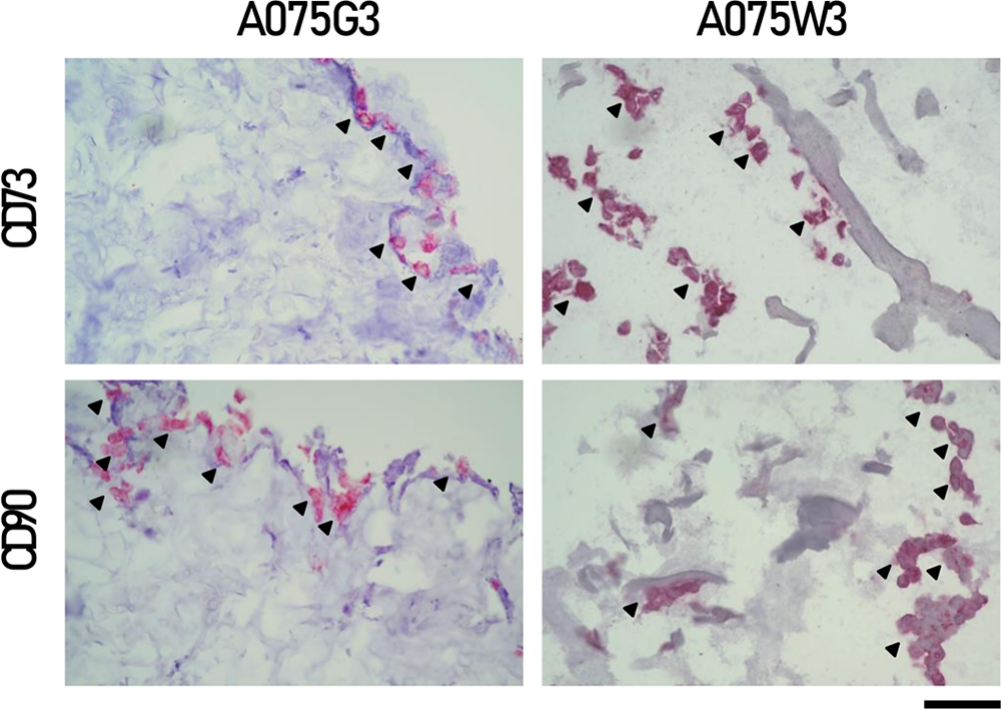
Immunohystochemical analyses of CD73 and CD90 in IVD cells combined with A075G3 and A075W3 millicylinders for 7 days. Representative images are shown; stained cells are indicated by arrows. Scale bar: 50 μm.

Another type of cells, human macrophages, were tested with millicylinders to evaluate their biological effect in terms of anti-inflammatory properties in a different culture condition represented by the Transwell culture system in which the millicylinders were placed in the upper chamber. As described in the Methodology section, human monocytes were differentiated into macrophages, polarized toward the M1 phenotype, exposed for 48 h to millicylinder stimuli, and assayed for the expression of CD80, a pro-inflammatory antigen. As shown in Figure 11, neither A075G3 nor A075W3 induced an upregulation of CD80 expression; indeed, A075W3 is likely capable of releasing factors into the culture medium that cause a moderate but significant reduction in CD80 levels.

**Figure 11:**
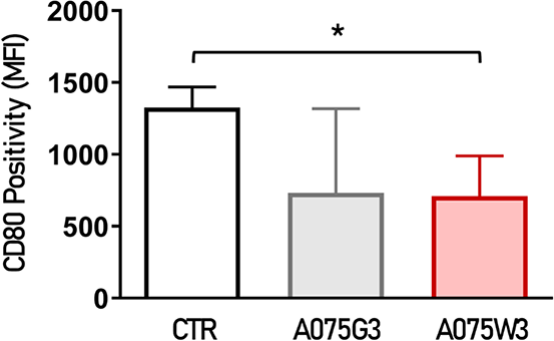
Human M1-polarized macrophages were exposed to A075G3 and A075W3 for 48 h. Positivity for CD80, a pro-inflammatory surface marker, was assessed by flow cytometry and expressed as mean fluorescence intensity (MFI) ± SD (*n* = 4 for CTR, A075G3 and A075W3). *p < 0.05 (A075W3 vs CTR).

## 5 Conclusions

Growing interest in incorporating DWJ, a natural polymer, into other biomaterials to fabricate high-performance scaffolds of various shapes and sizes has led to the development of multiple manufacturing strategies, including various chemical modifications of the constituent biomaterials. The primary purpose of the present report was not to compare different DWJ-based scaffold fabrication methods;^9,11,18,27–34^ we believe that such a comparison is still premature, particularly in light of the specificity of the different experimental models employed and the unique characteristics of the tissue damage to be repaired or the microenvironment to be regenerated. Instead, we focused on the numerous technical and methodological steps standardized after several experiments to produce and characterize a specific DWJ-based scaffold, which nevertheless remain highly versatile and adaptable to a wide range of tissue engineering applications. In our opinion, sharing the critical steps of a protocol like the one described here and enabling others to adopt and adapt it is essential to obtain informative biological assessments in subsequent studies. With this report, we aimed to provide a detailed and timely overview of the various steps of a protocol that can lead to the creation of millicylindrical scaffolds based on DWJ, potentially used to counteract joint degeneration in general, and intervertebral disc degeneration in particular. The data reported here are the results of numerous repeated tests/assays aimed at developing the ideal conditions for obtaining DWJ-based scaffolds capable of maintaining structural integrity, responsiveness to mechanical stimuli, and pro-anabolic properties over time. Specifically, we have sought to suggest methodological approaches useful for overcoming some critical issues associated with the bio inspired hydrogel, which is difficult to handle but in which we are investing due to its richness in pro-discogenic factors, which we have sought to preserve.

## Supplementary Material



## Notes

Hematoxylin/eosin staining of A075G3, A075W3 and native human IVD (PDF)
