# Supplementary material for: Methods and Technical Issues for Optimizing the Production of Hydrogels Containing Decellularized Wharton’s Jelly
Source: ACS Biomater Sci Eng. 2026 Feb 3;12(3):1446–58. doi: 10.1021/acsbiomaterials.5c02006 (PMC12976996; doi:10.1021/acsbiomaterials.5c02006)
Supplement: Supplementary file 1 [file ab5c02006_si_001.pdf]

Supporting information for:

Methods and technical issues for optimizing the production of hydrogels containing decellularized Wharton's jelly

Anna Chierici,† Giovanni D'Atri,† Cristina Manferdini, Elisabetta Lambertini, Gina Lisignoli, Roberta Piva, Claudio Nastruzzi,* and Letizia Penolazzi*

*** Corresponding Authors**

Letizia Penolazzi - *Department of Neuroscience and Rehabilitation, University of Ferrara, 44121 Ferrara, Italy*; Email: pnlmlt@unife.it

Claudio Nastruzzi - *Department of Chemical, Pharmaceutical and Agricultural Sciences, University of Ferrara, 44121 Ferrara, Italy;* Email: nas@unife.it

†A.C. and G.D'A. contributed equally to this work.

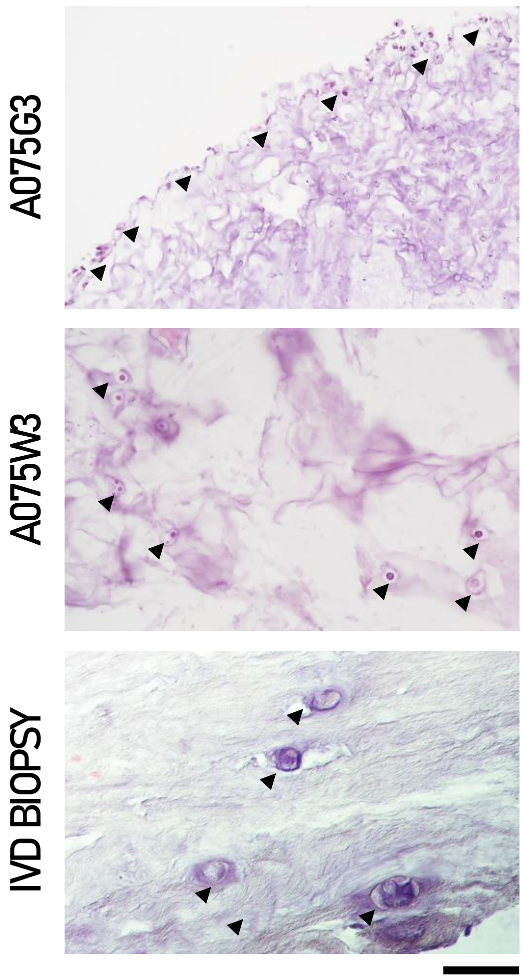

Supplementary Figure 1. Comparison of A075G3, A075W3 and native human IVD biopsy in terms of cell morphology and distribution. Representative images of histological hematoxylin/eosin staining are shown. Cells are indicated by arrows. Scale bar: 100 µm.
